# Remote Monitoring of the Performance Status and Burden of Symptoms of Patients With Gastrointestinal Cancer Via a Consumer-Based Activity Tracker: Quantitative Cohort Study

**DOI:** 10.2196/22931

**Published:** 2021-11-26

**Authors:** Alireza Ghods, Armin Shahrokni, Hassan Ghasemzadeh, Diane Cook

**Affiliations:** 1 School of Electrical Engineering and Computer Science Washington State University Pullman, WA United States; 2 Geriatrics / Gastrointestinal Oncology Service Memorial Sloan-Kettering Cancer Center New York, NY United States

**Keywords:** step count, performance status, symptom, wearable, activity tracker, gastrointestinal cancer, monitoring, cancer, gastrointestinal, burden

## Abstract

**Background:**

The number of older patients with gastrointestinal cancer is increasing due to an aging global population. Minimizing reliance on an in-clinic patient performance status test to determine a patient’s prognosis and course of treatment can improve resource utilization. Further, current performance status measurements cannot capture patients' constant changes. These measurements also rely on self-reports, which are subjective and subject to bias. Real-time monitoring of patients' activities may allow for a more accurate assessment of patients’ performance status while minimizing resource utilization.

**Objective:**

This study investigates the validity of consumer-based activity trackers for monitoring the performance status of patients with gastrointestinal cancer.

**Methods:**

A total of 27 consenting patients (63% male, median age 58 years) wore a consumer-based activity tracker 7 days before chemotherapy and 14 days after receiving their first treatment. The provider assessed patients using the Eastern Cooperative Oncology Group Performance Status (ECOG-PS) scale and Memorial Symptom Assessment Scale-Short Form (MSAS-SF) before and after chemotherapy visits. The statistical correlations between ECOG-PS and MSAS-SF scores and patients’ daily step counts were assessed.

**Results:**

The daily step counts yielded the highest correlation with the patients' ECOG-PS scores after chemotherapy (*P*<.001). The patients with higher ECOG-PS scores experienced a higher fluctuation in their step counts. The patients who walked more prechemotherapy (mean 6071 steps per day) and postchemotherapy (mean 5930 steps per day) had a lower MSAS-SF score (lower burden of symptoms) compared to patients who walked less prechemotherapy (mean 5205 steps per day) and postchemotherapy (mean 4437 steps per day).

**Conclusions:**

This study demonstrates the feasibility of using inexpensive, consumer-based activity trackers for the remote monitoring of performance status in the gastrointestinal cancer population. The findings need to be validated in a larger population for generalizability.

## Introduction

The number of gastrointestinal cancer cases is predicted to increase due to the aging population [1]. Moreover, patients in geographical areas with fewer services already experience health care disparities, while pandemic-related government restrictions such as stay-at-home orders resulted in fewer checkups [2,3]. A remote monitoring system can provide personalized care to larger populations without any geographical limitations. This study investigates the use of wearable activity trackers as an alternative to standard, in-person tests. In oncology, patients' performance status is a crucial factor in treatment decision-making and their prognosis. Tests such as the Eastern Cooperative Oncology Group Performance Status (ECOG-PS) scale [4,5] and the Memorial Symptom Assessment Scale-Short Form (MSAS-SF) [6] have been used to assess patients’ status. Although there is abundant evidence that patients' ECOG-PS and MSAS-SF scores correlate with cancer-related outcomes such as chemotherapy toxicity and response to treatment [7], both tests have limitations. The use of activity trackers to monitor patients can mitigate these limitations and provide a more accurate picture of patients’ status.

Although patients spend most of their time between cancer treatments at home, tests such as the ECOG-PS and MSAS-SF are conducted at clinic visits and do not provide a daily view of patients' performance status [8]. As a result, the tests' reliability and validity may be diminished due to the low agreement between clinicians, nurses, and patients on performance status ratings. A study by Ando et al [9], which included 206 patients with lung cancer, revealed that patients rate their ECOG-PS lower than oncologists and nurses. Similarly, Blagden et al [10] observed that oncologists and patients agreed about patients' ECOG-PS in only 50% of cases for 98 patients with lung cancer. Moreover, similar studies illustrate that interrater reliability decreases as patients' functional activity declines. Although interrater reliability was high between a clinical oncologist, a ward resident, and a medical officer for highly active patients [10], Mayer et al [11] found only 53%-61% agreement in ECOG-PS of patients with cancer in the palliative care setting.

Incorporating patient-generated health data can reduce bias and improve the accuracy of the patients' performance status tests. Electronic mobile activity trackers provide new methods for collecting and monitoring patients' daily activities and function in real settings. The feasibility of commercially available activity trackers has already been demonstrated for patients with other types of cancer [12]. As surveyed by Purswani et al [13], tracking the number of steps patients take is a key component of the evaluation of patients' health status in oncology. Perez et al [14] observed that a decrease in the number of daily steps among older patients with cancer is an indicator of chemotherapy toxicity. Gresham et al [15] demonstrated a strong correlation between average daily steps and ECOG-PS for patients with cancer. Although Soh et al [16] validated the use of a mobile care system for self-monitoring in patients with advanced gastrointestinal cancer, the utility of activity trackers in patients with gastrointestinal cancer is less explored. In this pilot study, we evaluate the correlations between patients’ ECOG-PS and MSAS-SF scores and their step counts. Further, we explore the best way to visualize the data to track daily fluctuations and monitor patients’ health status.

## Methods

### Overview

The development phase of this study began in February 2019. The Memorial Sloan Kettering Cancer Center Institutional Review Board authorized the conduct of this study in August 2019. Medical professionals were recruited from Memorial Sloan Kettering Cancer Center in New York, and the resulting team included oncologists, an oncology nurse specialist, oncology rehabilitation physicians, and a customer relationship management expert.

### Recruitment

Patients were eligible to participate in the study if they were aged ≥18 years, had gastrointestinal cancer, and started a new line of chemotherapy. Patients were excluded if they were using assistive devices such as a walker or a cane or were receiving concomitant radiation and chemotherapy. Additionally, patients needed to be enrolled in the study for at least seven days before starting the new chemotherapy line to allow for a proper baseline activity assessment. All patients gave their written consent to participate in the study.

### Technologies and Technique

Each participant was given a Misfit Shine AT fitness tracker (Misfit) after institutional review board approval and written informed consent. This particular model was selected after assessing various consumer-based activity trackers based on the following four characteristics:

1. No feedback provided. Patients should not receive any feedback regarding their step count, nor any positive or negative reinforcement in response to a high or low number of steps [17,18].
2. Long battery life. Patients should not need to remove the device to recharge it, which would potentially result in forgetting to put it back on again [19].
3. Waterproof. The activity tracker should be waterproof to allow patients to continue wearing it while showering.
4. Ability to act as an independent device. The activity tracker should be able to act as an independent device and not require synchronization with a cell phone.

The Misfit Shine exhibits all these characteristics and best fit our needs for this study. Misfit Shine has been validated for clinical use in prior studies [13,20,21]. For example, Ferguson et al [21] demonstrated a strong correlation between measurements obtained by the Misfit Shine and research-grade activity monitors. Furthermore, Mercer et al [22] observed a high acceptance rate of the Misfit Shine device among adults aged >50 years.

### Data Collection

A Misfit account was created for each patient and patients were instructed to wear the Misfit Shine on their nondominant wrist. The number of daily steps was recorded automatically in the app via Wi-Fi. Clinicians had access to the patients’ data on the administrator web page. An unidentified code was applied to each patient for security. It is important to mention that patients did not have access to their accounts in order to prevent them from reviewing their step count. Step count data were collected for each patient for 7 days prechemotherapy and 14 days postchemotherapy. A day with a step count >100 was referred to as a “full day of data collection,” a day with a step count <100 was referred to as a “partial day of data collection,” and a day with no step count recording was referred to as “no data collection.” Only patients with at least three full days of data collection during both the prechemotherapy and postchemotherapy periods were included in the final study.

### Step Count Assessment

A research study assistant collected patient data in two phases. C1D1 (cycle 1, day 1) indicates that the data were collected before the first cycle of chemotherapy (“prechemotherapy”). C2D1 (cycle 2, day 1) indicates that the data were collected after the first cycle of chemotherapy and before the second cycle (“postchemotherapy”). Patient data were collected 7 days before C1D1 and 14 days after C1D1. There was no intervention involved in the activity monitoring, and the data were collected after the completion of each cycle, not in real-time.

### Symptom Burden Assessments

Data on the presence and severity of symptoms were collected at baseline and at C2D1 by administering the MSAS-SF [6]. The MSAS-SF is a patient-rated instrument that evaluates 26 physical symptoms and the frequency of 4 psychological symptoms. Patients’ physical symptoms were assessed using a Likert scale ranging from 0 (not present) to 4 (very much). The frequency of psychological symptoms was rated from 1 (rarely) to 4 (almost constantly). The MSAS-SF comprises three subscales: the global distress index (GDI), physical symptom subscale (PHYS), and psychological symptom subscale (PSYCH). The MSAS-GDI assesses the average frequency of 4 psychological symptoms (sadness, irritability, nervousness, and anxiety) and 6 physical symptoms (lack of appetite, lack of energy, drowsiness, pain, constipation, and dry mouth). MSAS-PHYS is the average score of 12 physical symptoms: lack of appetite, pain, constipation, lack of energy, drowsiness, nausea, vomiting, dry mouth, change in taste, feeling bloated, dizziness, and weight loss. The MSAS-PSYCH assesses the average frequency of 6 psychological symptoms: anxiety, nervousness, sadness, difficulty sleeping, difficulty concentrating, and irritability. Finally, the total MSAS (TMSAS) score is the average score of all 32 physical and psychological symptoms.

## Results

### Patient Characteristics

**Figure 1:**
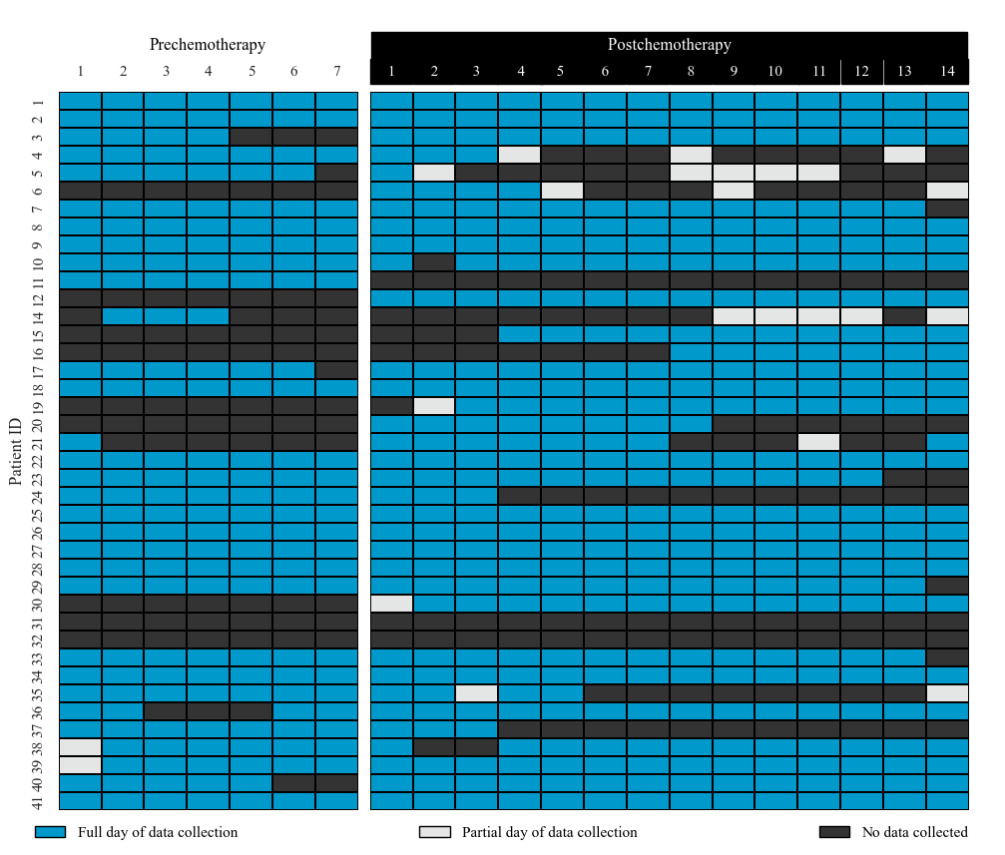
Completeness of patient data collection.

A total of 41 patients consented to the study, but one patient dropped out of the study because they decided to receive treatment at another institution. Only 27 patients (68%) had adequate activity tracker data, as shown in Figure 1. There were 17 males and 10 females, with a median age of 58 years (range 38-81 years). At baseline, patients had ECOG-PS scores of 0 (n=17, 63%) and 1 (n=10, 37%). The majority of patients were diagnosed with colon cancer (n=17) and were receiving metastatic chemotherapy (n=13). In this study, patients had lower TMSAS scores (mean 0.63, SD 0.37) compared to the broader cancer population (mean 0.77, SD 0.53) [16]. Patients also had lower scores on the MSAS-GDI, MSAS-PHYS, and MSAS-PSYCH compared to the broader cancer population. A lower MSAS score is an indicator of a low ECOG-PS score [23,24]. Additional information about patient characteristics is provided in Table 1. The patients’ step counts with and without adequate data at baseline are shown in Table 2 and Table 3.

**Table 1:** Demographic and clinical characteristics at baseline visit.

Demographics and characteristics		Values
**Gender, n (%)**		
	Male	17 (63)
	Female	10 (37)
Age (years), median (range)		58 (37-83)
**Marital status, n (%)**		
	Married	18 (66)
	Single	9 (34)
**Education, n (%)**		
	College graduate or higher	15 (55)
	Lower than college degree	12 (45)
**Chemotherapy types, n (%)**		
	Adjuvant	8 (30)
	Neoadjuvant	6 (22)
	Metastatic	13 (48)
**Smoking status, n (%)**		
	Ever	10 (27)
	Never	17 (63)
**Eastern Cooperative Oncology Group Performance Status** **score, n (%)**		
	0	17 (63)
	1	10 (37)
	>1	0 (0)
**Memorial Symptom Assessment Scale score, mean (SD)**		
	Global distress index subscale	0.63 (0.37)
	Physical symptom subscale	0.69 (0.52)
	Psychological symptom subscale	1.28 (0.75)
	Total Memorial Symptom Assessment Scale	0.63 (0.37)

**Table 2:** The daily mean, median, maximum, and minimum activity level pre- and postchemotherapy.

Treatment phases and days			Mean activity level	Median activity level	Maximum activity level	Minimum activity level
	**Prechemotherapy**					
		Day 1	0.713^a^	0.724^a^	0.269	0.572^a^
		Day 2	0.729^a^	0.779^a^	0.350	0.540^a^
		Day 3	0.857^a^	0.782^a^	0.773^a^	0.776^a^
		Day 4	0.820^a^	0.720^a^	0.875^a^	0.895^a^
		Day 5	0.842^a^	0.785^a^	0.558^a^	0.798^a^
		Day 6	0.720^a^	0.724^a^	0.372	0.632^a^
		Day 7	0.729^a^	0.692^a^	0.585^a^	0.584^a^
**Postchemotherapy**						
		Day 1	0.193	0.096	0.296	0.091
		Day 2	0.625^a^	0.556^a^	0.596^a^	0.563^b^
		Day 3	0.676^a^	0.591^a^	0.709^a^	0.406^b^
		Day 4	0.858^a^	0.793^a^	0.923^a^	0.361
		Day 5	0.894^a^	0.900^a^	0.805^a^	0.569^b^
		Day 6	0.913^a^	0.929^a^	0.817^a^	0.550^b^
		Day 7	0.885^a^	0.897^a^	0.835^a^	0.439^b^
		Day 8	0.716^a^	0.738^a^	0.630^a^	0.566^b^
		Day 9	0.549^a^	0.591^a^	0.421^b^	0.584^b^
		Day 10	0.862^a^	0.892^a^	0.738^a^	0.609^b^
		Day 11	0.829^a^	0.807^a^	0.822^a^	0.442^b^
		Day 12	0.863^a^	0.870^a^	0.744^a^	0.563^b^
		Day 13	0.858^a^	0.862^a^	0.776^a^	0.507^b^
		Day 14	0.725^a^	0.764^a^	0.642^a^	0.351

^a^*P*<.05.

^b^*P*<.001.

**Table 3:** The correlation of mean, median, maximum, and minimum activity levels pre- and postchemotherapy.

Activity levels	Prechemotherapy mean activity level	Prechemotherapy median activity level	Prechemotherapy maximum activity level	Prechemotherapy minimum activity level
Postchemotherapy mean activity level	0.839^a^	0.763^a^	0.794^a^	0.644^a^
Postchemotherapy median activity level	0.823^a^	0.774^a^	0.762^a^	0.695^a^
Postchemotherapy maximum activity level	0.792^a^	0.648^a^	0.838^a^	0.509^b^
Postchemotherapy minimum activity level	0.501^b^	0.605^b^	0.293	0.412^b^

^a^*P*<.05.

^b^*P*<.001.

### Step Count and Its Correlation With Performance Status

**Figure 2:**
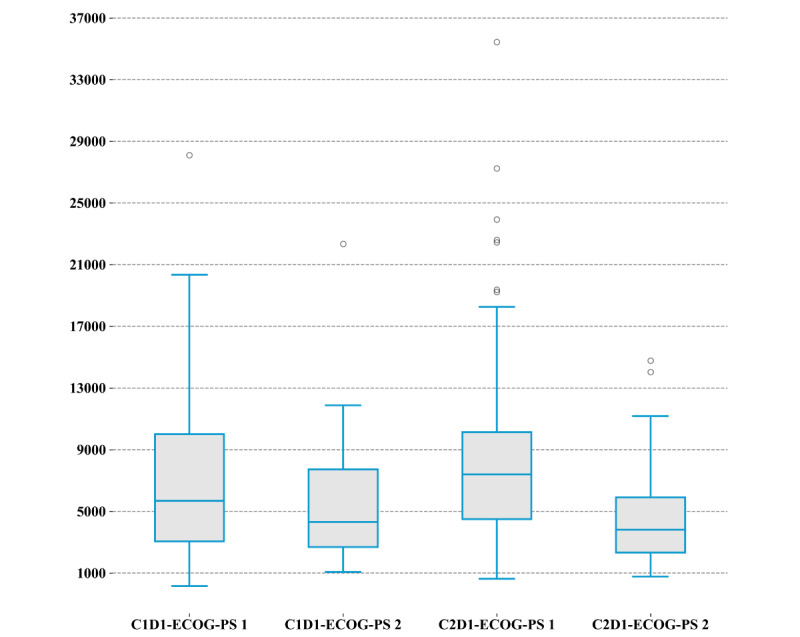
The step count per day by ECOG-PS score. C1D1 indicates that the data were collected before chemotherapy, and C2D1 indicates that the data were collected after chemotherapy. ECOG-PS: Eastern Cooperative Oncology Group Performance Status.

The overall average number of steps per day for all patients was 6290 before chemotherapy and 6325 after chemotherapy. The average step count prechemotherapy for patients with an ECOG-PS of 1 was 7023 steps per day, while patients with an ECOG-PS of 2 had an average step count of 5405 steps per day (Figure 2). The average step count at postchemotherapy for patients with an ECOG-PS of 1 was 8020 steps, while the average step count at C2D2 for patients with an ECOG-PS of 2 was 4448 steps. Although the correlation between both ECOG-PS categories at C1D1 was not significant (*P*=.06), there was a significant correlation at C2D1 (*P*<.001). The patients with an ECOG-PS of 0 had a higher median step count after chemotherapy. Conversely, the median step count for patients with an ECOG-PS of 2 decreases after chemotherapy. It is notable to mention that we did not find a significant correlation between either cancer type and number of steps or type of chemotherapy and number of steps.

### Effect of Chemotherapy on Patients’ Step Count

**Figure 3:**
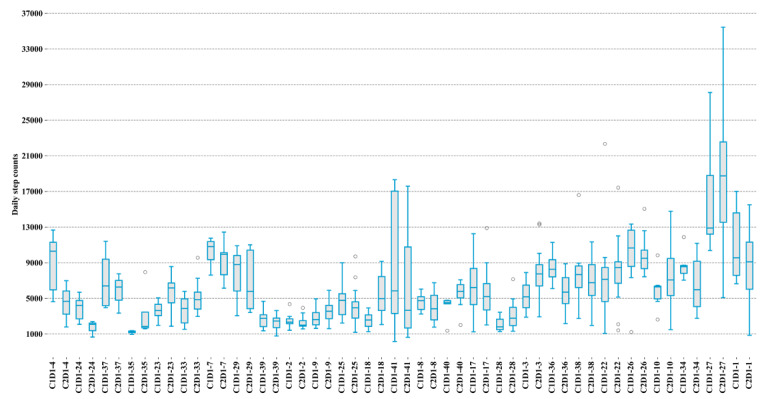
The daily step count of each patient before the first cycle of chemotherapy (labeled as C1D1) and after the first cycle of chemotherapy (labeled as C2D1).

The overall median number of steps walked by patients pre- and postchemotherapy was 4983 and 5480, respectively. Overall, the step count decreases after chemotherapy; the median difference between pre- and postchemotherapy for the cohort was 497 steps, and the IQR decreased from 5916 steps to 5119 steps postchemotherapy. Prechemotherapy, patients younger than 60 years of age walked more than patients older than 60 years of age (median number of steps 5618 versus 4738, *P*=1.4). This difference persisted postchemotherapy as well (median number of steps 5860 versus 4534, *P*=.002). Figure 3 illustrates the daily step count of patients before and after chemotherapy.

**Figure 4:**
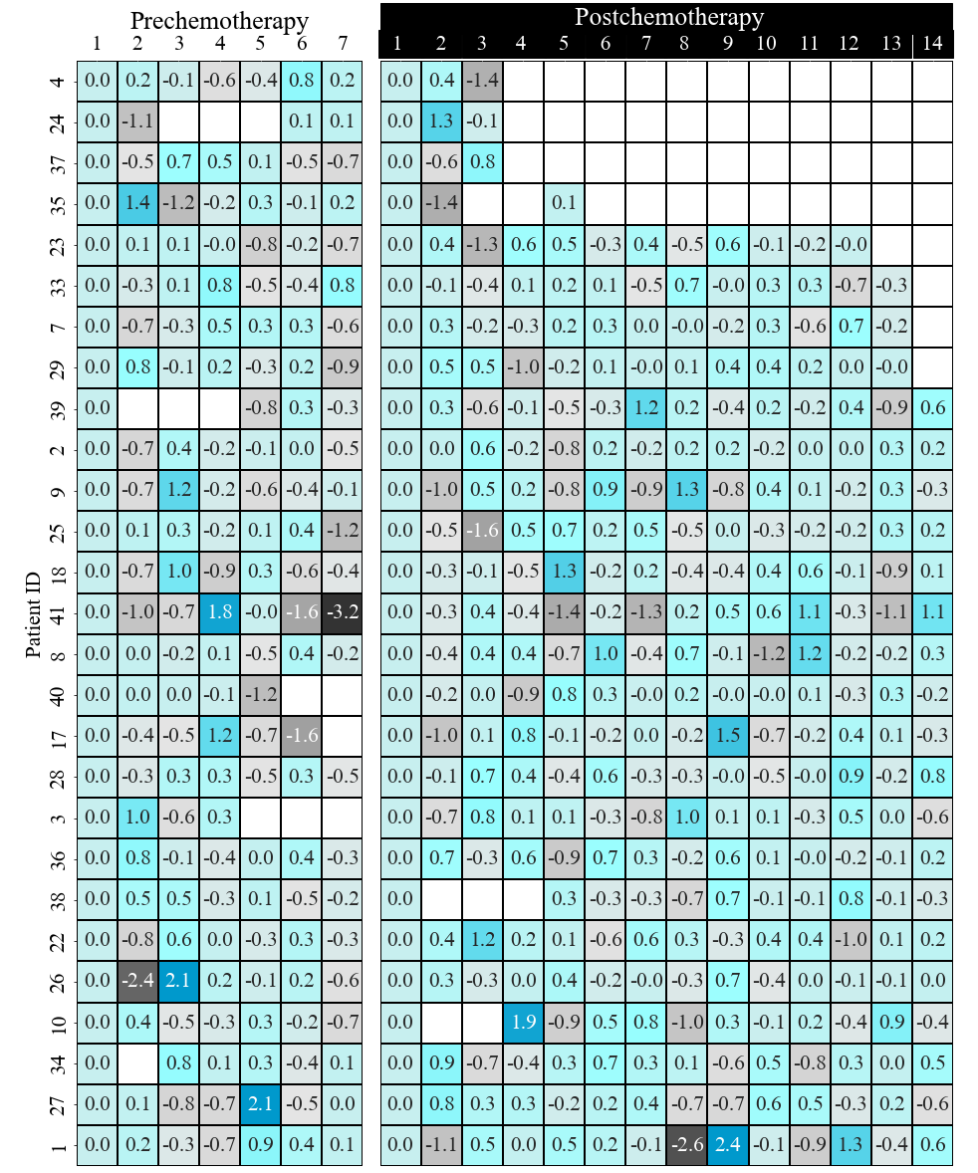
Step count volatility for each patient.

**Figure 5:**
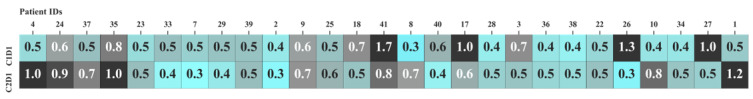
The annualized variance of the step counts of patients before (labeled as C1D1) and after (C2D1) the first cycle of chemotherapy.

We calculated the volatility of step counts pre- and postchemotherapy to illustrate the degree of behavior change, as follows:




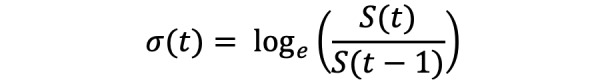




where *S(t)* is the number of steps at time *t*. A positive *σ(t)* shows an increase in step count compared to the previous day and a negative *σ(t)* indicates a decrease in step count compared to the previous day. Prechemotherapy, there were 120 days for which the step count increased compared to the previous day and 56 days for which the step count decreased compared to the previous day. Postchemotherapy, there were 222 days for which the step count increased compared to the previous day and 102 days for which the step count decreased compared to the previous day. Figure 4 displays the daily changes in step count for all patients. The annualized volatility of the step counts of each patient was calculated to explain the volatility of behavior change before and after chemotherapy, as follows:




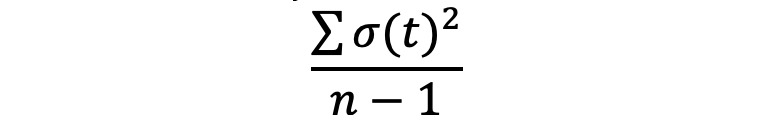




where *n* is the number of days with available data. The annualized variance in step count increased for 16 patients postchemotherapy, and decreased for 11 patients. Of the patients who experienced an increase in their annualized variance step count, 9 of them were patients with an ECOG-PS of 0 and 7 of them were patients with an ECOG-PS of 1. Among patients with an ECOG-PS of 1, 70% experienced an increase in the annualized variance step count compared to patients with an ECOG-PS of 0 (52%). Figure 5 illustrates the annualized variance step count of patients before and after chemotherapy.

### Step Count and Its Correlation With Burden of Symptoms

The median physical and psychological scores prechemotherapy were 0.53 (IQR 0.26-1.06) and 1.26 (IQR 0.66-1.86), respectively. The median GDI and TMSAS scores were 1.12 (IQR 0.64-1.56) and 0.66 (IQR 0.30-0.88), respectively. Patients’ symptom burden changed after chemotherapy. Patients had a median improvement of 0.18 for their GDI score, 0.09 on the TMSAS, and 0.26 for the psychological score, while the physical score did not change. In addition, 59% (16/27) had an improvement in their GDI and 62% (17/27) had an improvement in their TMSAS during the postchemotherapy phase. The rate of improvement for the cohort was 46% and 65% for the physical and psychological domains, respectively.

**Figure 6:**
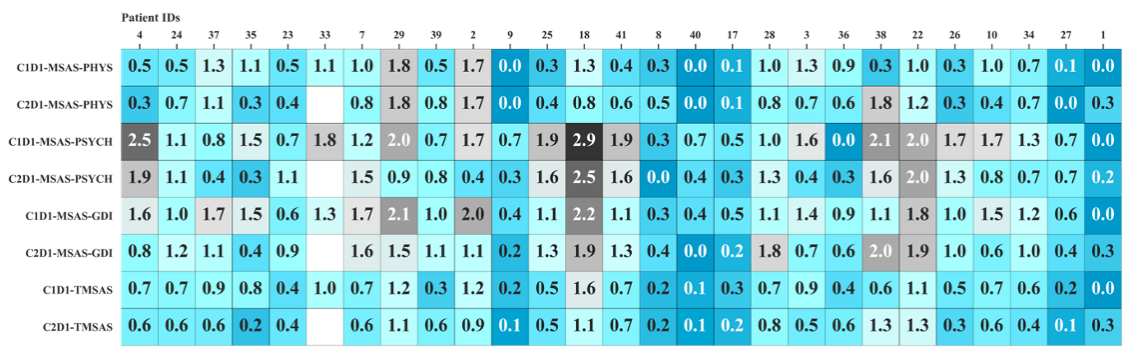
Patients' MSAS scores before and after the first day of the first cycle of chemotherapy (labelled as C1D1 and C2D1, respectively). GDI: global distress index; MSAS-SF: Memorial Symptom Assessment Scale-Short Form; PHYS: physical symptom subscale; PSYCH: psychological symptom subscale; TMSAS: total MSAS.

Those with an improvement in their GDI, TMSAS, and physical scores took more daily steps before and after chemotherapy compared to those with no improvement in these scores. The three patients who experienced an improvement in their GDI, physical, and TMSAS scores walked 6205, 5769, and 6239 steps before chemotherapy and 5788, 6216, and 5788 steps daily after chemotherapy. However, those with no improvement in their GDI, physical, and TMSAS scores walked 5032, 5436, and 5148 steps per day before chemotherapy, and 3934, 4562, and 4816 steps per day after chemotherapy. All MSAS scores of patients before and after chemotherapy are shown in Figure 6. Given the small sample size, the *P* value was not significant for any of these assessments.

### Feasibility and Acceptance of Activity Tracker

Only 13 of 40 patients did not have adequate data. There were 8 patients without adequate data prechemotherapy, 3 patients without adequate data postchemotherapy, and 2 patients with inadequate data both pre- and postchemotherapy. Of the collective 280 prechemotherapy days of the study cohort, there were 195 days (69%) with a “full day of data collection,” 2 days (1%) with a “partial day of data collection,” and 83 days (30%) with “no data.” Of the collective 560 postchemotherapy days of the study cohort, there were 405 days (72%) with a “full day of data collection,” 21 days (3%) with a “partial day of data collection,” and 134 days (25%) with “no data.” During the 7-day prechemotherapy phase, on average, patients had 5 days with a “full day of data collection.” During the 14-day postchemotherapy phase, patients had an average of 10 days with a “full day of data collection.” Patients with adequate activity tracker data were younger compared to those with inadequate data (median age 58 years versus 60 years, *P*=.59).

Out of 27 participants, only one participant had discomfort when wearing the activity tracker prechemotherapy; however, this person found it comfortable to wear the device postchemotherapy. In addition, two patients found it uncomfortable to wear the activity tracker postchemotherapy. The patient who experienced discomfort when wearing the activity tracker prechemotherapy had an increase in the number of steps taken postchemotherapy. In contrast, for the patients who had trouble with the device postchemotherapy, the number of steps decreased. The median satisfaction score pre- and postchemotherapy remained the same at 80.

## Discussion

This study investigated the feasibility of employing consumer-based activity trackers to monitor patients with gastrointestinal cancer undergoing chemotherapy. As shown in Figure 1, most patients wore their activity trackers during the 21-day study period. However, there was a drop-off in wearing the activity trackers at the end of each cycle. Previous studies illustrated a similar drop-off in the number of patients wearing their wearable devices [12,25]. As these results indicate, the length of study duration affects the amount of missing data. Thus, this increase in the amount of missing data and solutions to mitigate this problem need to be studied.

The study results indicate statically significant correlations between the number of steps patients take daily and two common performance status tests (ECOG-PS and MSAS-SF), which is consistent with earlier research findings [15]. These observations provide preliminary evidence supporting the clinical validity of using activity trackers in the care of patients with gastrointestinal cancer. As reported, patients with higher ECOG-PS scores experienced a higher volatility in their step count. Moreover, patients with a higher step count also had lower MSAS-GDI and TMSAS scores; this indicates that more active patients experience a lower burden of symptoms. These results suggest that physical activity could improve patients’ symptoms. Correspondingly, clinicians should promote physical activity in patients undergoing chemotherapy to keep patients’ symptoms under control.

We developed a steps volatility chart as a remote activity monitoring tool, as shown in Figure 4. Clinicians can easily track patients' daily activity levels by looking at the chart. The graph of patients' step volatility may be employed for interventions in a manner similar to other monitoring systems [26-28]. Use of the step volatility chart for cancer prevention and control and survivorship of patients should be studied in the future.

We believe patients' step counts, coupled with ECOG and MSAS scores, can help clinicians better understand patients' conditions. Activity tracker data provide a dynamic view of patients and could decrease the bias in patients' assessment tests. Although our study was limited by patient sample size, the number of monitored days, and our patients' performance status, we studied our patients in an uncontrolled environment outside clinical settings. In doing so, we illustrated the functionality of using wearable activity trackers to collect data in real life. The patients in this study tend to be healthier, with lower ECOG-PS scores, than the broader cancer population. Although this may limit the generalizability of our findings to a broader population, our results are in line with other studies on patients with severe conditions [12,13]. Our study's relatively healthy population demonstrates the usability of the wrist-worn activity tracker for this particular population.

In conclusion, the remote monitoring of patients' physical activity could decrease the cost of health care and provide a higher quality of health care to a broader population. Remote monitoring could revolutionize how we treat patients and help to provide health care for patients who live in remote areas without direct access to health care clinics or at times when doctors cannot see their patients in person. As a next step, we will collect data from a larger sample of patients with cancer with a broader range of ECOG-PS scores and find an approach that will encourage patients to use wearable activity trackers more regularly.

## Abbreviations

C1D1: cycle 1, day 1
C2D1: cycle 2, day 1
ECOG-PS: Eastern Cooperative Oncology Group Performance Status
GDI: global distress index
MSAS-SF: Memorial Symptom Assessment Scale-Short Form
PHYS: physical symptom subscale
PSYCH: psychological symptom subscale
TMSAS: total MSAS
